# Low sensitivity of conventional fungal agars in fungemia by *Rhodotorula mucilaginosa*: description of two cases

**DOI:** 10.1186/s12941-021-00427-w

**Published:** 2021-03-27

**Authors:** Carmen Alicia Garcia-Gutiérrez, María Soledad Cuétara-García, María Dolores Moragues, Jorge Ligero, Sara María Quevedo, María José Buitrago

**Affiliations:** 1 Department of Microbiology, Hospital Universitario Severo Ochoa, Leganés, Madrid, Spain; 2 Department of Nursing, School of Medicine and Nursing, University of the Basque Country UPV/EHU, Bilbao, Spain; 3 Mycology Reference Laboratory, Centro Nacional de Microbiología, Instituto de Salud Carlos III, Ctra Majadahonda-Pozuelo Km2, 28220 Majadahonda, Madrid, Spain

**Keywords:** Rhodotorula mucilaginosa, Fungemia, Specific culture media, Risk factors

## Abstract

**Background:**

Although most bloodstream yeast infections are caused by *Candida* spp., infections by rare or less common species have increased in recent years. Diagnosis of infections caused by these species is difficult due to the lack of specific symptoms and adequate diagnostic tools.

**Cases presentation:**

We describe two cases of fungemia by *Rhodotorula mucilaginosa* within a few months of each other, in a secondary Spanish hospital. In both cases, diagnosis was challenging. Blood subcultures in conventional fungal media were persistently negatives and the use of non-conventional fungal media was essential for isolating the yeasts and achieving a correct diagnosis. 1–3 beta-d-glucan detection and a panfungal PCR assay were helpful techniques to confirm the diagnosis

**Conclusion:**

It is highly important to establish an early diagnosis for fungemia. The process is challenging because often non-specific symptoms are presents. When yeasts grow in blood cultures other genera than *Candida* spp. could be the cause of infection. Patient risk factors should be assessed to incorporate alternative culture media and the available rapid diagnostic test, in order to provide an early recognition of the pathogen.

**Supplementary Information:**

The online version contains supplementary material available at 10.1186/s12941-021-00427-w.

## Background

Diagnosis of invasive fungal infections (IFI) is challenging due to a lack of specific symptoms and adequate diagnostic tools. While fungemia being the easiest one to identify via blood cultures, the sensitivity varies according to the technique used and the pathogen involved. Fungi are isolated in 10% of cases, 3.3% of them in mixed forms [1]. Although most bloodstream yeast infections are caused by *Candida* spp., recent years have seen an increase of infections by rare or less common yeast species such as *Trichosporon, Cryptococcus, Saccharomyces, Malassezia* and *Rhodotorula* spp [2]. In the described cases of fungemia by *Rhodotorula* spp., the main species involved is *Rhodotorula mucilaginosa* (also known as *Rhodotorula rubra* [3–6]). The mortality rate of fungemia by *Rhodotorula* spp. was reported as being 14.4% in a retrospective review [6]. In this report, fungemia not associated with central venous catheter (CVC) had a higher mortality rate than endocarditis and CVC fungemia (20% vs 14.2% vs 13.5%). This increased mortality is due to several reasons: (i) the population affected [7] (immunosuppressed patients and patients with risk factors such as CVC, peritoneal dialysis or parenteral nutrition), (ii) diagnostic difficulty and (iii) resistance to echinocandins [4, 5, 8–10]. To highlight the difficulty involved in achieving a correct diagnosis, we present two cases of fungemia due to *R. mucilaginosa* that occurred in a secondary hospital (350 beds) within a 3-month period where conventional fungal agars were useless and only potato dextrose agar was helpful to isolate the fungus.

## Cases presentation

### Case 1

A severely malnourished 49-year-old male with Crohn’s disease, treated with Imurel^®^ since 1999 and Infliximab^®^ since 2015, and a double-J catheter carrier due to hydronephrosis grade II/IV, received care at the emergency room of the Hospital Universitario Severo Ochoa (Leganes, Madrid) on November 20th, 2017 (day 0) due to severe hypomagnesemia (1 mg/dl). As a low-grade fever of 37.6 °C was found, urine and blood were extracted for cultures and he was discharged after biochemical correction. On day + 5, upon visualizing yeasts in two blood cultures (Versa TREK™ Automated Culture System, Thermo Scientific), the patient was called back to the hospital where he recorded a temperature of 37.4 °C without any other symptoms. Empirical treatment with caspofungin^®^ was instituted and control blood cultures as well as a urine culture were performed. The positive blood cultures were subcultivated in blood and chocolate agar and in different fungal media: sabouraud dextrose with antibiotics (SCG2 BioMerieux), BBL CHROMagar Candida Medium (Becton Dickinson) and potato dextrose agar plates, all incubated at 35 °C. On day + 6, eye fundus study, transthoracic echocardiogram and thoracoabdominal radiology were all normal. On day + 8, a blood sample was tested for biomarkers 1–3 beta-d-glucan (BDG) (Fungitell^®^, Assoc. Cape Cod Inc. USA) and CAGTA (Candida albicans IFA IgG, Vircell^®^ kit assay, Granada, Spain) with negative results. The isolation of yeasts only occurred in blood, chocolate and potato dextrose agar plates, while the other agar plates remained sterile after five days. *R. mucilaginosa* was identified phenotypically and by MALDI-TOF on day + 9, and antifungal treatment was changed from Caspofungin (275 mg total doses) to AmBisome^®^iv (130 mg/dl). On day + 10, due to a marked worsening of the abdominal pelvic computed tomography (CT) with bilateral renal lithiasis, the double J was replaced and the immunosuppressors were suspended temporarily until resolution of the fungemia. After 16 days with AmBisome^®^iv, the patient was asymptomatic and blood and urine cultures for yeasts were all negative, as were serial biomarkers.

### Case 2

An 80-year-old man with type 2 diabetes mellitus, normotensive chronic hydrocephalus and a ventriculoperitoneal shunt performed on November 7, 2017, entered hospital 1 month later (62 days) due to fever of unknown origin and to confusional state. He was treated with empirical intravenous (iv) ceftazidime and iv vancomycin. On day-28 the patient was discharged. Three months later, on February 8th (day 0) he returned to the hospital due to febrile peaks and neurological deterioration, presenting an increase in acute phase reactants (C-reactive protein 23 mg/L, leukocytosis 13,010 cells/mm^3^ with neutrophilia 83.9%). Blood and urine samples for bacterial cultures were extracted. Yeasts were detected in two blood cultures on day + 5 (Versa TREKTM Automated Culture System, Thermo Scientific). AmBisome^®^ iv 375 mg was administered followed by doses of 200 mg per day. Oral 5-fluorocytosine was also administered at a concentration of 1750 mg/12 h. Blood was extracted for biomarkers study (BDG and CAGTA).

The positive blood cultures were subcultivated in blood and chocolate agar, and in the following fungal media: sabouraud dextrose with antibiotics (SCG2 BioMerieux), BBL CHROMagar Candida Medium (Becton Dickinson), and potato dextrose agar plates, all at 35 °C. On day + 6, as meningitis was suspected, a cranial CT scan revealed no new alterations, and a lumbar puncture was performed. The cerebrospinal fluid (CSF) was suggestive of chronic subacute meningitis (proteins 73 mg/dl, glychorrhachia 48 mg/dl, 45 cells/mm3, 98% mononuclear) with negative conventional fungal and bacterial cultures. Due to clinical worsening, antifungal treatment was changed to iv anidulafungin. Yeast isolation only occurred in blood agar, chocolate agar and potato dextrose agar, while the other agar plates remained sterile after five days. The species *R. mucilaginosa* was phenotypically identified by MALDI-TOF on day + 7, and AmBisome was reinstated maintaining the anidulafungin for two more days. CSF study was positive for BDG (85.64 pg/ml) and for a panfungal PCR that targets the ITS1 region of the rDNA [11]. Briefly, the assay was performed in a BioRad CFX96 unit (BioRad, Madrid, Spain). The assay was a Real Time PCR method that used the universal primers ITS1 and ITS2 [12] and the kit Sensimix SYBR No-ROX (Bioline, Ecogen, Madrid, Spain). The PCR reaction (20 µL) contained 0.8 µM each primer and 4.5 mM MgCl_2_. The conditions for the PCR reaction were: 95 °C for 10 min; 45-cycle as follows: 10 s at 95 °C, 5 s at 54 °C, and 30 s at 72 °C. A melting program from 65 °C to 97 °C was performed. Melting curves were analyzed to discriminate unspecific amplifications from true amplicons. Agarose gel electrophoresis was performed at 1.5% in order to verify the quality of the amplified product. Amplified fragments were sequenced and compared with the ITS nucleotide sequence database available in the Mycology Reference Laboratory (which contains more than 10,000 distinct sequences) and with the GenBank database (https://www.ncbi.nlm.nih.gov/genbank/). The sequence matched those of genus *Rhodotorula* and the species *R. mucilaginosa* (90%). The moderate quality of the sequence did not permit us to achieve the species level but confirmed the genus *Rhodotorula.* The alignment with the sequences that best matched at GenBank is shown in the Additional file 1: Figure S1.

In spite of a total dosage of 1775 mg of AmBisome the patient died at day + 14.

## Discussion and conclusions

*Rhodotorula* is a basidiomycetous yeast genus that belongs to the family Cryptococcaceae. These yeasts are universally distributed in nature (air, soil, water of lakes and oceans) and as saprophytes in skin, nails, and the respiratory, gastrointestinal and urinary tracts of humans. Their microbiological identification is based on the production of a carotenoid pigment, which gives them salmon-pink to coral-red appearance in initially smooth and moist colonies, which become rough and dull with time. Yeasts are ovoid to spherical (2–3 × 5–7 µm) occurring singly or in chains, occasionally showing multilateral budding. Neither mycelium nor pseudomycelium is produced. They do not ferment carbohydrates and are urease positive. *Rhodotorula mucilaginosa*, *Rhodotorula glutinis* and *Rhodotorula minuta* are the most clinically relevant species of the genus [8].

Although generally these species are considered as being non-pathogenic, they can cause disease in both immunocompromised and immunocompetent patients, with *R. mucilaginosa* being responsible for more than 70% of infections [13]. The first report of fungemia by *Rhodotorula* spp. appeared in 1960 [14]. Since then it has emerged as an opportunistic pathogen associated with indwelling CVCs and immunosuppresion (solid and hematological neoplasm patient) [6, 7]. Broad-spectrum antibiotics, corticosteroids, hyperalimentation, surgery, chronic renal failure, diabetes mellitus, granulocytopenia and damage to mucosa and skin have been suggested as predisposing factors for *Rhodotorula* infections [6].

In this article, two cases of proven fungemia by *R. mucilaginosa* diagnosed in a three months period in a 350 bed secondary hospital are described. Both cases corresponded to hospitalized patients with known risk factors, no previous history of azole treatment and are no time-related.

**Fig. 1:**
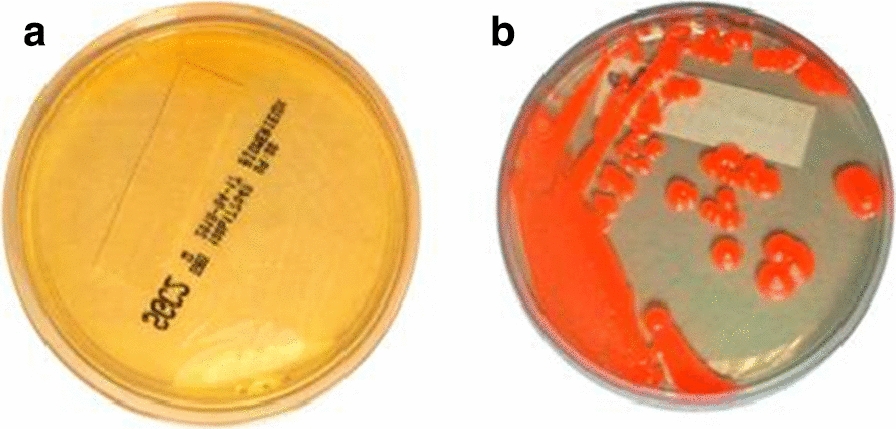
Subcultures of blood culture: **a** *Rhodotorula mucilaginosa* on Sabouraud agar; **b** and on potato dextrose agar

Sensitivity of conventional cultures in fungemia is around 50%. In patients with suspected fungal infection, guidelines recommend performing from two to four blood cultures and even incorporating specific bottles [15]. In this two cases, after the vision of yeasts in Gram, subcultures in blood and chocolate agar were performed as well as in the following fungal media: sabouraud dextrose agar with antibiotics (SCG2 BioMerieux), BBL CHROMagar Candida Medium (Becton Dickinson) and potato dextrose agar medium. In both cases, the isolation of yeast only occurred in blood, chocolate and potato dextrose agar, while the other agar plates remained sterile (Fig. 1), which leads us to recommend the use of various culture media to increase sensitivity of fungal isolation. Both strains were identified by using MALDI-TOF MS (Bruker Daltonics, Billerica, MA, USA) and API32C auxonogram (profile 546175011) [5] (BioMerieux, France). The lack of growth of *Rhodotorula* isolates in two common, commercially available, fungal media (sabouraud dextrose agar with antibiotics (SCG2 (BioMerieux) and BBL CHROMagar Candida Medium (Becton Dickinson) is not due either to the strain or to the media as in the other subcultures the strains were able to grow. The source of lack of grow could be found in the two step process involving blood culture in bottle and subsequent subculture on these specific agar plate media. In fact, these media are routinely used for the isolation of *Rhotorula* spp. in other superficial and deep samples. Specifically, in the last five years this yeast has been isolated in 19 samples (skin biopsies, ascitic and peritoneal liquids, blood, otics and ungueal samples) using these same media. The characteristics of the strains, the low fungal burden or the blood culture media could be in the origin of the lack of grow.

The Versa TREKTM Automated Culture System has been successfully used for *Candida* spp. however specific studies with *Rhodotorula* spp. have not being performed. Deeper studies should be done to confirm this findings.

As expected, MIC values (µg/mL; Sensititre^®^ Yeast One panel-Trek Diagnostic Systems, Cleveland, OH, USA) were high for echinocandins (> 8) and fluconazole (> 256); variable for all other azoles tested: voriconazole (4 and 8), posaconazole (2), itraconazole (1 and 2); and low for amphotericin B (2 and 0.25) and 5-fluorocytosine (0.125 and < 0.006), respectively. The molecular mechanism behind the reduced susceptibility to echinocandins among basidiomycetous yeasts remains poorly understood [4, 5, 9, 10]. Carotenoid pigments produced by *R mucilaginosa* strains could play a role in imparting resistance against echinocandins but has not being proven.

As other authors [9] and guidelines [16] reported previously: (i) amphotericin B should be the treatment of choice in cases of *R. mucilaginosa* infection, (ii) 5-fluorocytosine could be considered as an adjuvant treatment given its good in vitro performance and (iii) withdrawl of CVC if present is essential [16] given the ability of this yeast to adhere to catheters or other prosthetic devices by producing biofilms [5]. The length of treatment of *Rhodotorula* fungemia is variable in the literature, ranging from 14 to 41 days [13].

Although *R. mucilaginosa* contains BDG in its wall [17], only five cases have been reported in which this biomarker was used for diagnosis [18]. Serum levels of BDG in both cases described here were low (4.4 pg/ml and 67 pg/ml, respectively) as also previously reported by other authors [18, 19]. However, the detection of BDG in cerebrospinal fluid in the second patient was very useful to support the etiology of meningitis. The reason why obtaining low serum levels of BDG in these species is unknown but it might be because the capsule prevents releasing this polysaccharide to serum, or because of limited or transient fungemia.

Panfungal PCR, based on the amplification of the ITS1 region of ribosomal DNA, was performed only in case 2, obtaining a positive result and highlighting the usefulness of this type of technique as complementary to conventional diagnostic techniques. However, the quality of the sequence did not allow for identification at the species level, probably because the CSF is not the most suitable sample for a panfungal assay.

An early and adequate treatment with the antifungal of choice as well as the elimination of predisposing factors (replacement of the double J and temporary suspension of the immunosuppressors) led to a clarification of yeasts in blood and to a clinical cure without sequels of the first patient. Unfortunately, this was not enough in the second case, mainly due to a more precarious basal situation and the fact that the infection was disseminated with meningitis (positive results of panfungal PCR and CSF BDG).

In conclusion, a rapid identification of fungi causing fungemia is a key issue for an adequate therapy. When yeasts grow in blood cultures, we must consider that genera other than *Candida* spp such as *Malassezia*, *Trichosporon*, *Cryptococcus* and *Rhodotorula* may be involved in the infection. Patient risk factors should guide us to incorporate alternative culture media such as Dixon agar for *Malassezia* (patients with parenteral lipid nutrition), media supplemented with catecholamines or coffee for *Cryptococcus* (in HIV-patient), or potato dextrose agar for *Rhodotorula* (in vascular catheterization and leukaemia) or *Trichosporon* (in leukaemia patients and prophylaxis with oral polyenes in ICU). The isolation and recognition of rare yeasts in invasive infections, as well as the detection of intrinsic antifungal resistance, imply a working overload in the mycology laboratory. Timely identification and rapid antifungal therapy is essential to reducing the morbidity and mortality of the patient.

## Supplementary Information

**Additional file 1: Figure S1**: Alignments obtained in GenBank with the sequences for both strands (overlapping was not successful) for the amplicon obtained by using the panfungal PCR in Case 2. A) Alignment with the sequence obtained by using primer ITS1 B) Alignment with the sequence obtained by using primer ITS2.

## Data Availability

The datasets during and/or analysed during the current study available from the corresponding author on reasonable request.
