# Supplementary material for: Low sensitivity of conventional fungal agars in fungemia by Rhodotorula mucilaginosa: description of two cases
Source: Ann Clin Microbiol Antimicrob. 2021 Mar 27;20:21. doi: 10.1186/s12941-021-00427-w (PMC8005224; doi:10.1186/s12941-021-00427-w)
Supplement: Supplementary file 1 — Additional file 1: Figure S1: Alignments obtained in GenBank with the sequences for both strands (overlapping was not successful) for the amplicon obtained by using the panfungal PCR in Case 2. A) Alignment with the sequence obtained by using primer ITS1 B) Alignment with the sequence obtained by using primer ITS2. [file 12941_2021_427_MOESM1_ESM.docx]

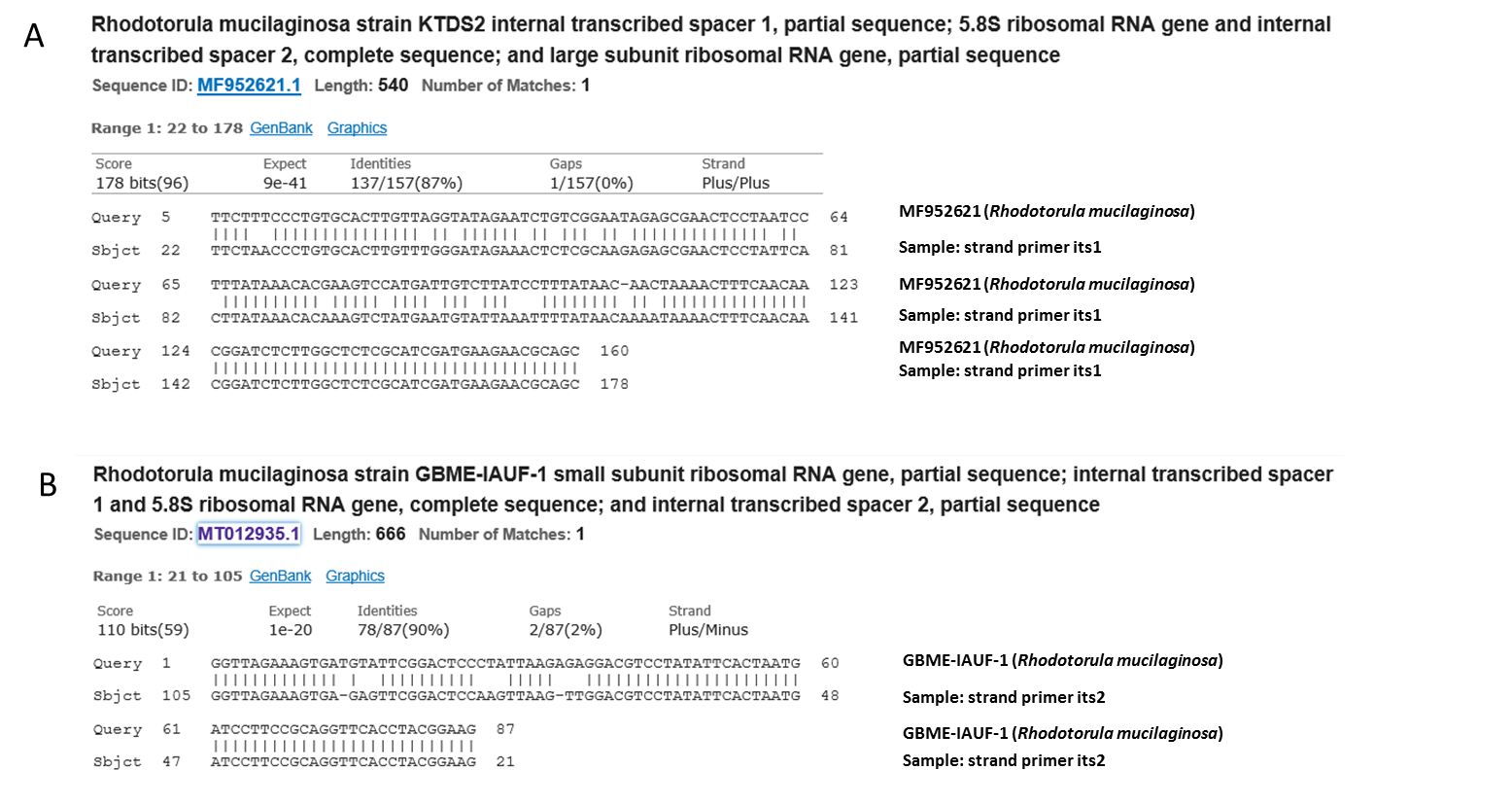


**Figure S1**: Alignments obtained in GenBank with the sequences for both strands (overlapping was not successful) for the amplicon obtained by using the panfungal PCR in Case 2. A) Alignment with the sequence obtained by using primer ITS1 B) Alignment with the sequence obtained by using primer ITS2
